# Atypical cutaneous mycobacteriosis caused by *M. fortuitum* acquired in domestic environment^☆^^☆☆^

**DOI:** 10.1016/j.abd.2019.06.012

**Published:** 2020-03-13

**Authors:** Dimitri Luz Felipe da Silva, Letícia dos Santos Valandro, Paulo Eduardo Neves Ferreira Velho, Andréa Fernandes Eloy da Costa França

**Affiliations:** Department of Medical Clinic, Faculdade de Ciências Médicas, Universidade Estadual de Campinas, Campinas, SP, Brazil

Dear Editor,

A previously healthy caucasian woman, 54 years old, came to dermatology outpatient clinic with an erythematous nodulocystic lesion, about 2 cm, with no drainage ostium, located on the dorsal surface of the 4th finger; associated with pain, edema and warmth (Fig. 1). The injury arised four days after local trauma while cleaning her home bathroom. She denied fever, or systemic symptom. There was no improvement despite the use of oral antibiotics and corticosteroids. The hypothesis of pheohyphomycosis, sporotrichosis and atypical mycobacteriosis were considered. Biopsy of the lesion and culture of the liquid content were performed. The anatomopathological analysis showed an organized chronic inflammatory process, occupying the entire thickness of the dermis, but without the presence of fungi and acid-fast bacilli – AFB (Fig. 2). *Mycobacterium fortuitum* was isolated from sample culture on Middlebrook 7H12. Serologies for HIV, hepatitis B and C, and syphilis were all negative. After confirmation of the etiologic agent, treatment with clarithromycin (1 g/day) and levofloxacin (1 g/day) was implemented, with posterior change of this last medication to sulfamethoxazole-trimethoprim (1200 mg/240 mg every 12 h), due to gastrointestinal intolerance, leading to complete regression of lesion after 6 months (Fig. 3).

**Figure 1:**
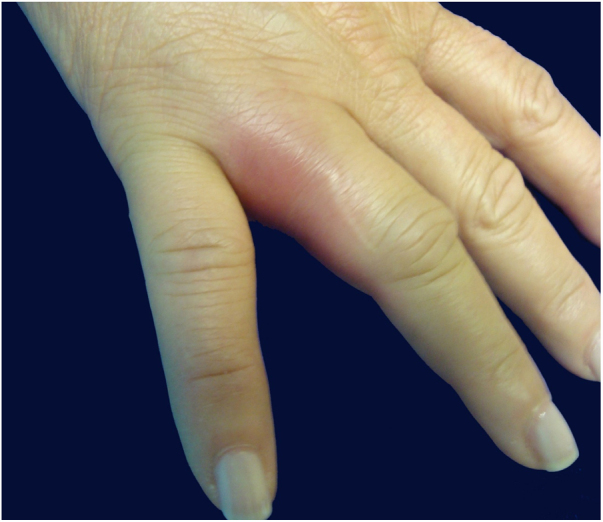
Right hand: edema and erythema on the fourth finger.

**Figure 2:**
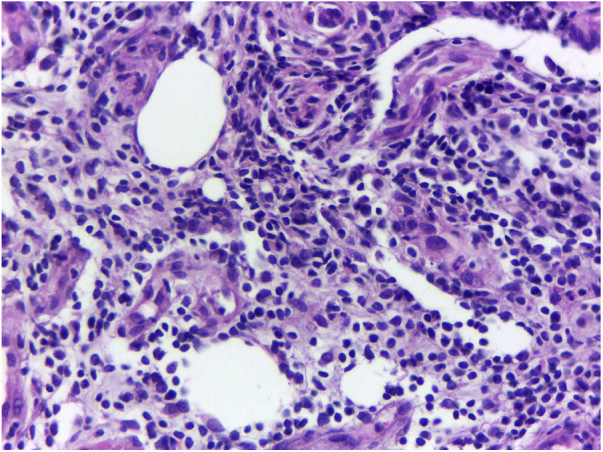
Chronic and organized inflammatory process, without detection of acid-fast bacilli and fungi (Hematoxylin & eosin, ×40).

**Figure 3:**
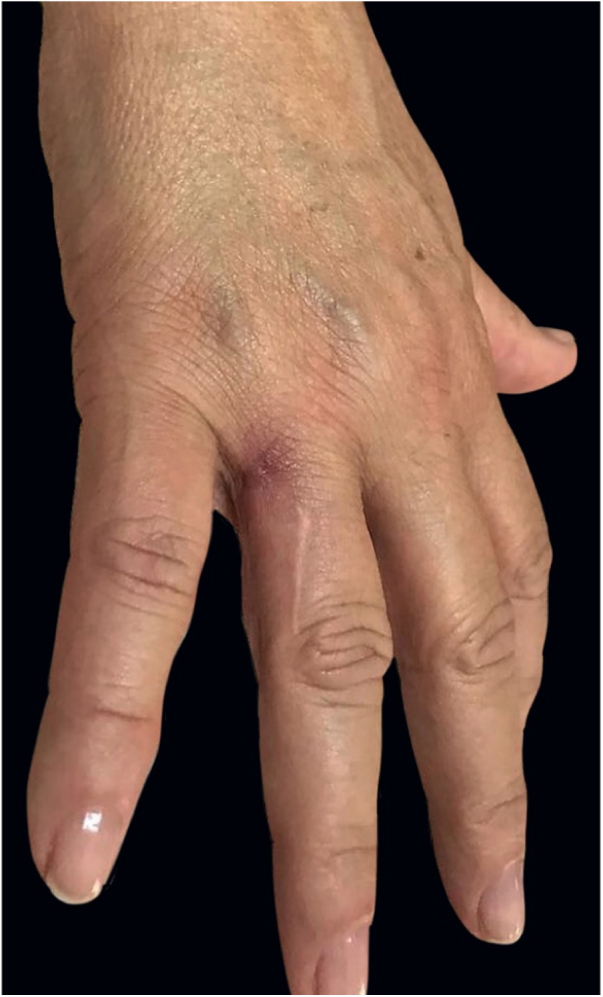
Regression of inflammation after antibiotic treatment.

Atypical mycobacteria, also known as MOTT (mycobacteria other than tuberculosis), are acid-fast bacilli with slow growth in culture and very peculiar behavior. MOTT may be saprophytic or found in animals, water and moist places. Atypical mycobacterioses correspond to 10% of mycobacterial infections and preferentially affect immunocompromised individuals.^1^ The rapidly growing mycobacteria (RGM), known by their one-week culture growth, can be found in various sites. The most relevant species are: *M. fortuitum*, *M. chelonae*, and *M. abscessus. M. fortuitum* is related with hospital infections in immunocompromised patients, leading to pulmonary, soft tissue and bone infections. Cutaneous involvement is more related to postoperative situations and invasive cosmetic procedures.^2^ The present case corresponds to cutaneous infection by *M. fortuitum* in an immunocompetent patient, acquired at home, probably by a trauma in an humid area. Differential diagnosis is made with swimming-pool granuloma, caused by *M. marinum*, due to the circumstances in which the infection was acquired, but culture allowed to the definitive etiology. The diagnosis of atypical mycobacterioses is made through the isolation of the agent in culture, since radiological, histopathological and clinical examinations are often inconclusive. The history of long-standing infection, without improvement after different treatments, can lead to clinical suspicion. The follow-up includes long-term broad-spectrum antibiotic treatment. The macrolide group in combination with quinolones is one of the most recommended regimens, sometimes requiring surgical intervention.^3^, ^4^ The present report reinforces the importance in considering atypical mycobacterioses among the differential diagnoses of traumatic cutaneous lesions, especially when they tend to chronicity.

## Authors’ contributions

Dimitri Luz Felipe da Silva: Approval of the final version of the manuscript; conception and planning of the study; elaboration and writing of the manuscript; obtaining, analysis, and interpretation of the data; critical review of the literature; critical review of the manuscript.

Letícia dos Santos Valandro: Conception and planning of the study; critical review of the literature.

Paulo Eduardo Neves Ferreira Velho: Intellectual participation in the propaedeutic and/or therapeutic conduct of the studied cases.

Andréa Fernandes Eloy da Costa França: Approval of the final version of the manuscript; elaboration and writing of the manuscript; intellectual participation in the propaedeutic and/or therapeutic conduct of the studied cases.

## Financial support

None declared.

## Conflicts of interest

None declared.
